# Analyses of Compact *Trichinella* Kinomes Reveal a MOS-Like Protein Kinase with a Unique N-Terminal Domain

**DOI:** 10.1534/g3.116.032961

**Published:** 2016-07-13

**Authors:** Andreas J. Stroehlein, Neil D. Young, Pasi K. Korhonen, Bill C. H. Chang, Paul W. Sternberg, Giuseppe La Rosa, Edoardo Pozio, Robin B. Gasser

**Affiliations:** * Faculty of Veterinary and Agricultural Sciences, The University of Melbourne, Parkville, Victoria 3010, Australia; † Yourgene Bioscience, Shu-Lin District, New Taipei City 23863, Taiwan; ‡ Howard Hughes Medical Institute, Chevy Chase, MD 20815-6789; § Division of Biology and Biological Engineering, California Institute of Technology, Pasadena, California 91125; ** Istituto Superiore di Sanità, 00161 Rome, Italy

**Keywords:** parasitic worms, *Trichinella*, kinome, protein kinases, protein annotation

## Abstract

Parasitic worms of the genus *Trichinella* (phylum Nematoda; class Enoplea) represent a complex of at least twelve taxa that infect a range of different host animals, including humans, around the world. They are foodborne, intracellular nematodes, and their life cycles differ substantially from those of other nematodes. The recent characterization of the genomes and transcriptomes of all twelve recognized taxa of *Trichinella* now allows, for the first time, detailed studies of their molecular biology. In the present study, we defined, curated, and compared the protein kinase complements (kinomes) of *Trichinella spiralis* and *T. pseudospiralis* using an integrated bioinformatic workflow employing transcriptomic and genomic data sets. We examined how variation in the kinome might link to unique aspects of *Trichinella* morphology, biology, and evolution. Furthermore, we utilized *in silico* structural modeling to discover and characterize a novel, MOS-like kinase with an unusual, previously undescribed N-terminal domain. Taken together, the present findings provide a basis for comparative investigations of nematode kinomes, and might facilitate the identification of Enoplea-specific intervention and diagnostic targets. Importantly, the *in silico* modeling approach assessed here provides an exciting prospect of being able to identify and classify currently unknown (orphan) kinases, as a foundation for their subsequent structural and functional investigation.

The phylum Nematoda (roundworms) includes a wide range of parasitic and free-living species with extensive biological and genetic diversity. Based on recent molecular studies, nematodes can be separated into multiple classes, including the Chromadorea and the Enoplea (Blaxter *et al.* 1998; Roberts and Janovy 2009; Blaxter and Koutsovoulos 2015). Within the latter class, the genus *Trichinella* forms a complex of at least twelve species and genotypes. These parasites infect a range of different host animals and have a worldwide distribution (Pozio 2007). Hosts become infected through the ingestion of muscle tissue containing first-stage larvae (L1s). In the gut, L1s are released from muscle cells, invade the small intestinal mucosa, and undergo a series of rapid moults (within ∼30 hr) to develop into male and female adults (Despommier 1993). Fertilized female worms release L1s into the lymph lacteals within ∼1 wk; these larvae are then disseminated via the blood stream into striated muscles, where they penetrate individual muscle cells. Over the course of 15–20 d, the host cell is transformed into what is known as a “nurse cell”. The parasite-host-cell-complex is surrounded by a collagen capsule for some (encapsulated) species, including *T. spiralis*; however, this capsule is absent from other (nonencapsulated) taxa, such as *T. pseudospiralis* (see Despommier 1993, 1998; Pozio *et al.* 2001). This intracellular environment allows *Trichinella* larvae to survive for up to 20 yr until ingested by a susceptible host (Pozio and Darwin Murrell 2006; Pozio and Zarlenga 2013).

The life cycles of *Trichinella* spp. differ markedly from those of most other parasitic nematodes in several respects. First, *Trichinella* L1s are unique in that they are adapted to survive inside host cells. Second, these parasites do not have free-living stages in their life cycle, and the adult female worms release live L1s, in contrast to many other parasitic nematodes which release eggs into the environment (Roberts and Janovy 2009). Third, the biological diversity within the *Trichinella* complex reflects considerable genetic variability among taxa, and different host affiliations and geographic distributions (Pozio 2007; Korhonen *et al.* 2016). The underlying molecular mechanisms responsible for these variable traits and habitats are still relatively poorly understood (Pozio and Zarlenga 2013).

Recently, advanced sequencing and bioinformatic technologies have enabled the characterization of the genomes and transcriptomes of all individual *Trichinella* taxa (Mitreva *et al.* 2011; Korhonen *et al.* 2016). The reported draft genomes for *T. spiralis* and *T. pseudospiralis* are 50 Mb and 49.2 Mb in size and encode 14,745 and 12,699 protein-coding genes, respectively (Korhonen *et al.* 2016). These genomic and transcriptomic data sets represent an invaluable resource for addressing some vexed questions surrounding the molecular biology of members of this species complex, in particular cellular signaling mechanisms, some of which have been investigated with regard to their involvement in remodeling of the host cell (Capo *et al.* 1998; Despommier 1998; Gounaris *et al.* 2001) and/or other host-parasite interactions (Arden *et al.* 1997; Smith *et al.* 2000; Gounaris *et al.* 2001).

In general, most signaling cascades rely heavily on protein kinases, which are important enzymes that phosphorylate one or more substrate proteins, leading to the downstream activation or inactivation of molecular signaling partners (Cohen 2000; Ubersax and Ferrell 2007). This process plays a central role in a wide range of biological processes, including the regulation of transcription, cell cycle, differentiation, and apoptosis (Cohen 2000; Ubersax and Ferrell 2007). Protein kinases can be classified into nine key groups, families, and subfamilies, based on sequence similarity in the catalytic kinase domain and functional domain architecture. A 10th group (“atypical”) consists of diverse protein kinases that show limited or no structural similarity to the protein kinase-like fold, but are active protein kinases (Hanks *et al.* 1988; Hanks and Hunter 1995; Manning 2005). Although many protein kinases are relatively conserved and often assume essential functions in all eukaryotes, some subsets are expanded in and/or specific to particular taxa (Manning 2005). For example, there are major expansions of several groups/families in the kinome of the free-living nematode *Caenorhabditis elegans* (see Plowman *et al.* 1999; Manning 2005), with some expansions specific to this nematode, and others that appear to be shared by a range of nematode species excluding *T. spiralis* (see Desjardins *et al.* 2013).

An initial assessment of a draft genome of *T. spiralis* (see Mitreva *et al.* 2011) suggested a substantial reduction in the protein kinase complement (kinome) of this species compared with the draft kinomes of other parasitic and free-living nematodes (Desjardins *et al.* 2013). Whether this apparent reduction might associate with the life style and unique biology of *T. spiralis* or with an incomplete genomic assembly (*cf*. Desjardins *et al.* 2013) is not yet clear. To address some of these questions, we used transcriptomic and genomic data sets for the two best-studied representatives of *Trichinella*, namely *T. spiralis* and *T. pseudospiralis* (see Korhonen *et al.* 2016) to (i) define, curate, and compare the protein kinase complements of these two species, (ii) explore quantitative and qualitative diversification of protein kinases in the *Trichinella* complex in comparison with other nematodes, and (iii) discuss how variation in kinase complements might link to the uniqueness of encapsulated and nonencapsulated species.

## Materials and Methods

### Defining kinomes

We used published genomic and transcriptomic data of representatives of encapsulated (*T. spiralis*; designated T1) and nonencapsulated (*T. pseudospiralis*; designated T4.1) *Trichinella* species (Korhonen *et al.* 2016), as well as a published kinase sequence data set (Desjardins *et al.* 2013) predicted from independent *T. spiralis* genomic data (Mitreva *et al.* 2011). We selected data sets of these two species as representatives because of the high quality of respective genomic and transcriptomic assemblies (Korhonen *et al.* 2016) and the extent of experimental work conducted on *T. spiralis* and *T. pseudospiralis* (see Pozio and Darwin Murrell 2006; Pozio and Zarlenga 2013).

First, from a total of 14,745 (*T. spiralis*) and 12,699 (*T. pseudospiralis*) inferred amino acid sequences (Korhonen *et al.* 2016), we predicted protein kinase candidates using InterProScan v.5.15.54 (Jones *et al.* 2014), employing information from domain-matches against the databases Pfam v.27.0 (Sonnhammer *et al.* 1997), PANTHER v.9.0 (Mi *et al.* 2013) and SUPERFAMILY v.1.75 (Gough *et al.* 2001). We selected the longest protein sequence of each predicted kinase, and assessed kinase candidates with incomplete or unusual domain architectures by comparisons with known kinase domain combinations, as reported in InterProScan and KinBase (http://kinase.com/web/current/kinbase/; accessed: May 1, 2016), and by literature searches.

Second, we identified groups of orthologs among the kinase sequences of *T. spiralis* and *T. pseudospiralis* using the program OrthoMCL v.2.0.4 (Li *et al.* 2003), applying a BLAST E-value cut-off of 10^−5^ and a stringent similarity cut-off of 80%. We then mapped “ungrouped” (*i.e.*, without an ortholog) protein sequences to the genomic scaffolds of the other species using the program BLAT v.34x12 (Kent 2002). Using the query sequences as templates, we then improved/complemented individual gene structures (intron–exon boundaries) by mapping to respective genomic regions using the program Exonerate (Slater and Birney 2005), employing the “multi-pass suboptimal alignment” parameter and the “protein2genome:bestfit” model. If complete open reading frames (ORFs) could not be identified, we matched both the coding sequence - inferred using Exonerate - and the amino acid template with a *de novo*-assembled transcript (Korhonen *et al.* 2016) using BLASTn v.2.2.28+ (E-value ≤ 10^−30^; Camacho *et al.* 2009) and BLAT, respectively. Then, we mapped matching transcripts (≥ 80% identity, with ≥ 80% of the original sequence length aligned) to genomic scaffolds to define their gene structures.

Third, we created pairwise global alignments of individual amino acid query sequences and all inferred heterologous sequences thereof employing the program EMBOSS Needle v.6.4.0.0 (Rice *et al.* 2000), retaining the sequence with the best alignment (spanning ≥ 80% of the query sequence).

Fourth, we classified all curated sequences using the program Kinannote (Goldberg *et al.* 2013), excluding partial sequences of < 200 amino acids in length. Sequences not classified using Kinannote were assessed for homology to those represented in well-curated kinomes of *C. elegans* (see Manning 2005) and *Homo sapiens* (see Manning *et al.* 2002b) using OrthoMCL (BLAST E-value ≤ 10^−5^; sequence similarity ≥ 80%); such sequences were assigned to a particular kinase family/subfamily if all *H. sapiens* and/or *C. elegans* sequences in an OrthoMCL cluster had the same family/subfamily classification. If sequences could not be assigned using this approach, domain architecture information (for atypical protein kinases; aPKs) or phylogenetic analysis (for eukaryotic protein kinases; ePKs) was used to classify them.

### Phylogenetic analysis

We used the catalytic domains [Pfam identifier PF07714 for tyrosine kinases (TK) and receptor guanylate cyclases (RGC); PF00069 for all other kinase groups] of all ePK sequences, in order to construct multiple sequence alignments for individual groups using the program hmmalign within the package HMMER v.3.1b1 (http://hmmer.janelia.org/). Then, we constructed phylogenetic trees (Bayesian inference) using the program MrBayes v.3.2.2 (Ronquist *et al.* 2012), employing a mixture of models with fixed rate matrices to calculate posterior probabilities. To construct majority rule trees, 1,000,000 trees were generated, of which every 100th tree was sampled after discarding the first 25% of trees as burn-in. Trees were drawn and annotated using the programs FigTree v.1.4.1 (http://tree.bio.ed.ac.uk/software/figtree/) and Inkscape (http://www.inkscape.org/en/), respectively.

### Functional and structural annotation of kinase sequences

We functionally annotated the curated kinase sequences by integrating information from InterProScan [gene ontology (GO) terms, Pfam, PANTHER, and SUPERFAMILY identifiers] and then assigned kinase sequences to biochemical pathways based on matches (BLASTp v.2.2.28+; E-value ≤ 10^−5^; Camacho *et al.* 2009) to the KEGG database (release August 27, 2014; Kanehisa and Goto 2000). The subcellular localization of kinases was inferred using the program MultiLoc2 (release October 26, 2009; Blum *et al.* 2009). Three-dimensional structures of select kinases were predicted using the program I-TASSER v.4.4 (Roy *et al.* 2010); structures were visualized and superimposed using the MatchMaker function within the program UCSF Chimera v.1.9 (build 39798; Pettersen *et al.* 2004).

### Data availability

Supplemental Material, Figure S1 contains high-resolution figures of trees representing the phylogenetic relationship of ePK sequences between *T. spiralis* and *T. pseudospiralis*. Table S1 and Table S2 list data and information on the kinase complements for *T. spiralis* and *T. pseudospiralis*, respectively, including information on orthologs/homologs, amino acid sequence identities and similarities, excretory/secretory predictions, functional annotations (domains/families/superfamilies), and amino acid sequences. Table S3, Table S4, Table S5, Table S6, Table S7, Table S8, Table S9, and Table S10 contain sequence identifiers describing the OrthoMCL clusters among *T. spiralis*, *T. pseudospiralis*, *C. elegans*, and *H. sapiens*, as shown in Figure S2. Genomic and transcriptomic sequences used in this work have been published previously (*cf*. Korhonen *et al.* 2016) and are available from the NCBI BioProject database under accession code PRJNA257433.

## Results

### The protein kinase complements of T. spiralis and T. pseudospiralis

**Figure 1:**
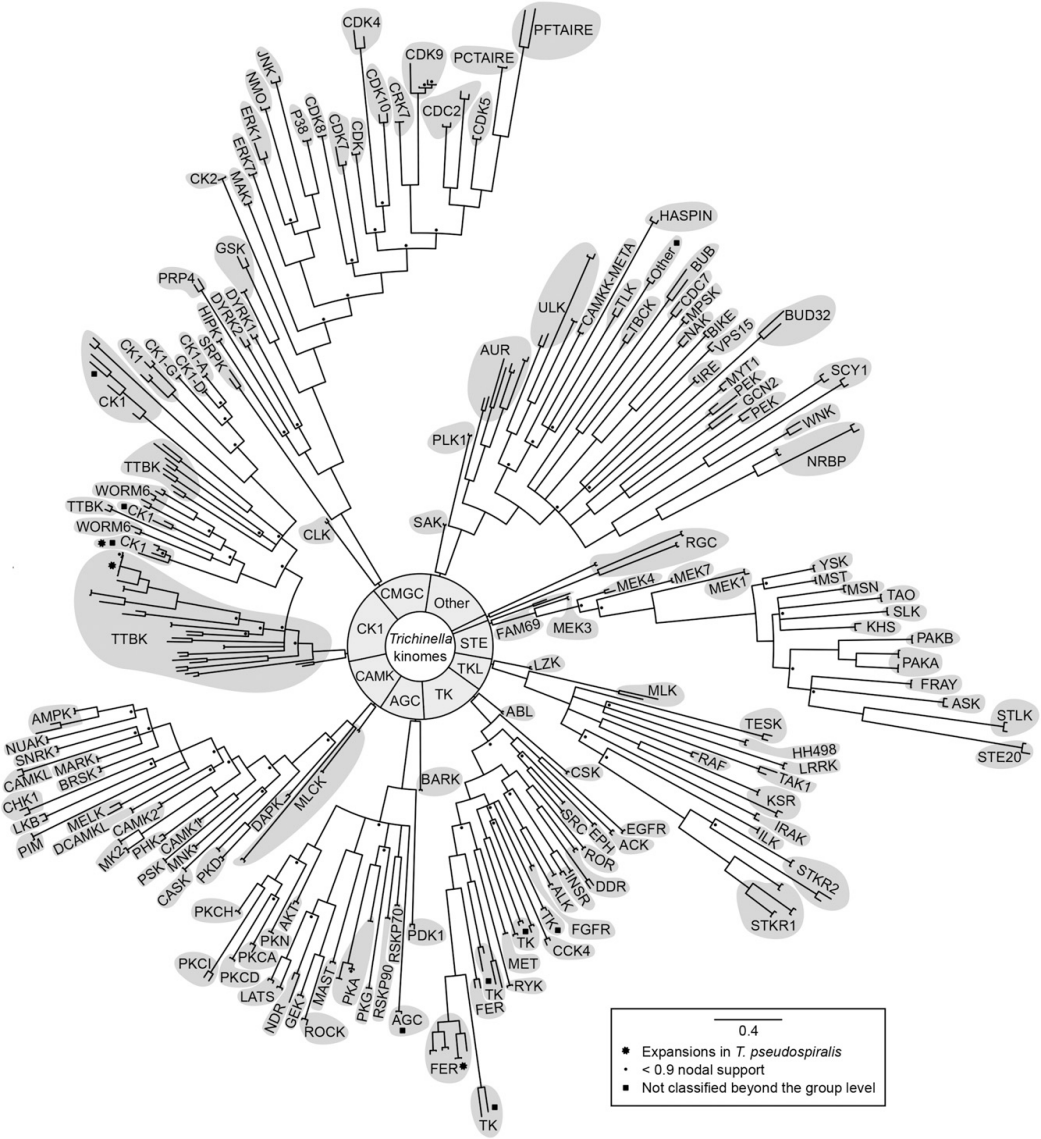
Phylogenetic analyses of eukaryotic protein kinases (ePKs) of *Trichinella spiralis* and *T. pseudospiralis*. Phylogenetic trees (Bayesian inference) were constructed based on alignment of amino acid sequences representing individual kinase groups. High-resolution figures of individual trees, including nodal support values and sequence identifiers, are given in Figure S1.

Employing our integrated bioinformatic workflow, we defined, curated, and annotated the kinase complements of *T. spiralis* and *T. pseudospiralis*. The kinomes of *T. spiralis* and *T. pseudospiralis* represented 226 and 232 kinases, respectively, of which 205 and 212 sequences were classified as ePKs (Table 1). For both species, the kinase complement represented all nine currently recognized groups; 193–198 sequences (93–94%) were assigned to 79 distinct families, and 93 kinase sequences (44–45%) could be classified into 81 subfamilies (Table 1). However, 12 and 14 ePKs (5.3–6%) could not be classified beyond the group level for *T. spiralis* and *T. pseudospiralis*, respectively, and were thus assigned to the groups AGC (*n* = 1), “Other” (*n* = 1), CK1 (*n* = 5–7), and TK (*n* = 5) (Figure 1 and Figure S1).

**Table 1:** Eukaryotic protein kinases (ePKs) of *Trichinella spiralis* (T1) and *T. pseudospiralis* (T4.1)

Groups						Families		Subfamilies	
Name	T1	T4.1	*Ce*	*Hc*	*Hs*	T1	T4.1	T1	T4.1
AGC	21	21	29	41	63	11 (20; 95%)	11 (20; 95%)	12 (13; 62%)	12 (13; 62%)
CAMK	26	26	40	60	74	12 (26; 100%)	12 (26; 100%)	11 (13; 50%)	11 (13; 50%)
CK1	33	39*^a^*	83	61	12	3 (28; 85%)	3 (32; 82%)	3 (3; 9%)	3 (3; 8%)
CMGC	28	28	48	43	61	8 (28; 100%)	8 (28; 100%)	20 (22; 79%)	20 (22; 79%)
Other	31	31	67	46	83	18 (30; 97%)	18 (30; 97%)	9 (11; 35%)	9 (11; 35%)
RGC	3	3	27	28	5	1 (3; 100%)	1 (3; 100%)	0 (0; 0%)	0 (0; 0%)
STE	18	18	24	27	47	4 (18; 100%)	4 (18; 100%)	15 (16; 89%)	15 (16; 89%)
TK	29	30*^a^*	84	56	90	16 (24; 83%)	16 (25; 83%)	1 (1; 3%)	1 (1; 3%)
TKL	16	16	15	24	43	6 (16; 100%)	6 (16; 100%)	10 (14; 88%)	10 (14; 88%)
Totals	205	212	417	386	478	79 (193; 94%)	79 (198; 93%)	81 (93; 45%)	81 (93; 44%)

The numbers of kinases in individual groups for *T. spiralis*, *T. pseudospiralis*, *C. elegans* (*Ce*; Manning 2005), *H. contortus* (*Hc*; Stroehlein *et al.* 2015) and *H. sapiens* (*Hs*; Manning *et al.* 2002b). For *Trichinella* species, the numbers of unique families and subfamilies and the numbers and percentages of kinases assigned to them (in brackets) are shown.

a Differences in the number of kinases between *T. spiralis* and *T. pseudospiralis*.

For both *Trichinella* species, most protein kinase families or subfamilies (*n* = 112) contained a single ePK representative (Table 2, Table S1, and Table S2); the remainder (*n* = 26) contained two to six ePK representatives, with the exception of the TTBK family (*n* = 19 and 23 for *T. spiralis* and *T. pseudospiralis*, respectively). Both species encoded all “core” ePKs (*cf*. Goldberg *et al.* 2013), except for a RAD53 kinase ortholog (CAMK group), which was also absent from all other recognized species or genotypes of *Trichinella* (see Korhonen *et al.* 2016). Other kinase families, members of which are not universally conserved among eukaryotes, were also absent, including members of the NEK family (Quarmby and Mahjoub 2005) as well as two growth factor receptors, VEGFR and PDGFR (Manning *et al.* 2002a). Furthermore, a homolog of the TTK kinase, MPS-1, was not detected in any of the currently recognized *Trichinella* taxa, supporting a previous proposal that this kinase is not encoded in the *T. spiralis* genome (Desjardins *et al.* 2013).

**Table 2:** Number of representatives in eukaryotic protein kinase (ePK) families and subfamilies of *Trichinella spiralis* and *T. pseudospiralis*

Number of ePK Families/Subfamilies	Families/Subfamilies	Number of Representatives (*T. spiralis*/*T. pseudospiralis*)
112	See Figure 1, Table S1, and Table S2	1/1
17	ALK, AMPK, CDC2, CDK9, CK1, DDR, GSK, INSR, KSR, LATS, MK2, NRBP, PAKA, PEK, PKD, SCY1, STKR2	2/2
5	MLCK, PKA, RGC, STKR1, ULK	3/3
2	AUR, WORM6	4/4
1	FER	5/6
1	TTBK	19/23

In addition to ePKs, we identified and curated 21 and 20 atypical protein kinases (aPKs) for *T. spiralis* and *T. pseudospiralis*, respectively, and assigned them to 17 families/subfamilies (Table 3). Both species have single-copy genes encoding ABC1-A, ABC1-B, Alpha, DHS-27, DNAPK, FRAP, PI4K, RIO1, RIO2, RIO3, TAF1, and TRRAP kinases. Other aPK families/subfamilies, including bromodomain kinases, pyruvate dehydrogenase kinases, ataxia-telangiectasia mutated (ATM)-related kinases, and uncharacterized nuclear-hormone receptor-related kinases, are encoded by multiple (*n* = 2–3) genes (Table 3, Table S1, and Table S2).

**Table 3:** Atypical protein kinases (aPKs) of *Trichinella spiralis* (T1) and *T. pseudospiralis* (T4.1)

Family/Subfamily	T1	T4.1	*Hc*	*Ce*	*Hs*	Description	Domains Present*^a^*
ABC1-A	1	1	1	1	2	Activity of bc1 complex kinase A	PF03109 (ABC1 family)
							SSF56112 (Protein kinase-like)
ABC1-B	1	1	2	1	2	Activity of bc1 complex kinase B	PF03109 (ABC1 family)
							SSF56112 (Protein kinase-like)
Alpha	1	1	1	1	6	Alpha kinase	PF02816 (Alpha-kinase family)
							PTHR14187 (Alpha kinase/elongation factor 2 kinase)
							SSF56112 (Protein kinase-like)
BRD	2*^b^*	1	5	1	4	Bromodomain	PF00439 (Bromodomain)
							SSF47370 (Bromodomain)
DHS-27*^c^*	1	1	14*^d^*	0	0	Uncharacterized oxidoreductase	SSF56112 (Protein kinase-like)
							IPR012877 (Uncharacterized oxidoreductase)
PDHK	2	2	1	1	1	Pyruvate dehydrogenase kinase	PTHR11947 (Pyruvate dehydrogenase kinase)
							SSF56112 (Protein kinase-like)
PI4K*^c^*	1	1	0	0	0	Phosphatidylinositol 4-kinase	PF00454 (Phosphatidylinositol 3-and 4-kinase)
							PTHR10048 (Phosphatidylinositol kinase)
							SSF56112 (Protein kinase-like)
ATM-related	5	5	7	5	6	Ataxia telangiectasia mutated-related (including ATM, ATR, DNAPK, FRAP, SMG1, TRRAP)	PTHR11139 (Ataxia telangiectasia mutated (ATM)-related)
							PF00454 (Phosphatidylinositol 3- and 4-kinase)
							SSF56112 (Protein kinase-like)
RIO1	1	1	1	1	1	Right open reading frame kinase 1	PF01163 (RIO1 family)
							PTHR10593 (Serine/threonine-protein kinase RIO)
							SSF56112 (Protein kinase-like)
RIO2	1	1	1	1	1	Right open reading frame kinase 2	PF01163 (RIO1 family)
							PF09202 (RIO2, N-terminal)
							SSF56112 (Protein kinase-like)
RIO3	1	1	1	1	1	Right open reading frame kinase 3	PF01163 (RIO1 family)
							PTHR10593 (Serine/threonine-protein kinase RIO)
							SSF56112 (Protein kinase-like)
TAF1	1	1	2	1	2	Transcription initiation factor TFIID subunit 1	PTHR13900 (Transcription initiation factor TFIID)
							PF00439 (Bromodomain)
							SSF47370 (Bromodomain)
NHRR*^c^*	3	3	14*^d^*	0	0	Uncharacterized nuclear-hormone receptor-related	SSF56112 (Protein kinase-like)
							PTHR23020 (uncharacterized nuclear hormone receptor-related)
							PF07914 (DUF1679)
Totals	21	20	37*^d^*	17	38		

aPKs were classified using Kinannote, supporting domain annotation from InterProScan and clusters of homologs with *H. contortus* (*Hc*), *H. sapiens* (*Hs*), and *C. elegans* (*Ce*; retrieved from http://kinase.com/web/current/kinbase/)

a Complete domain annotations are given in Table S1 and Table S2.

b aPK families with differences in the number of kinases between *T. spiralis* and *T. pseudospiralis*.

c Novel kinase families with putative protein kinase activity.

d In *H. contortus* (*Hc*), 14 unclassified sequences have both the DHS-27 domain and the NHRR domain.

A pairwise comparison of both ePKs and aPKs of *T. spiralis* and *T. pseudospiralis* revealed 212 kinase sequences to be single-copy orthologs (Figure 1, Table S1, and Table S2); 14 and 20 sequences represented clusters of multiple homologs in *T. spiralis* and *T. pseudospiralis*, respectively. A global pairwise comparison of orthologous kinase sequences of the two species had a mean identity of 85% and a similarity of 89%. Despite this high identity/similarity for the majority of kinases, several, small kinase expansions (Figure 1 and Figure S1, C and H) within the families TTBK (CK1 group) and FER (group TK) were evident for *T. pseudospiralis*.

### Functional annotation of Trichinella kinomes

To annotate all 458 identified kinase sequences from both species, we predicted protein function (GO terms), biochemical pathways (KEGG), and protein domains. Most kinase sequences were predicted to assume one or more of the following functions: “protein phosphorylation” (GO:0006468; *n* = 413), “protein kinase activity” (GO:0004672; *n* = 409), “ATP-binding” (GO:0005524; *n* = 346), and “protein-binding” (GO:0005515; *n* = 96). Approximately half of all sequences (*n* = 106–108; 47%) were assigned to one or more biological (KEGG) pathways. Interestingly, although no RGC kinases were linked to environmental information processing, as they are in other nematodes (Manning 2005; Ortiz *et al.* 2009; Adachi *et al.* 2010; Stroehlein *et al.* 2015), 66 kinases in all other groups were (AGC, *n* = 13; Atypical, *n* = 3; CAMK, *n* = 11; CK1, *n* = 3; CMGC, *n* = 7–8; Other, *n* = 2; STE, *n* = 10; TK, *n* = 10–11; and TKL, *n* = 6). In contrast, for both *Trichinella* species, the smallest number of kinases (*n* = 8) was associated with the KEGG category “metabolism.” The numbers of sequences assigned to the remaining KEGG categories “organismal systems,” “cellular processes,” and “genetic information processing” ranged from 18 to 63 (Table S1 and Table S2).

The prediction of functional domains showed that 447 sequences contained a protein kinase-like domain (IPR011009; SSF56112), 324 a protein kinase domain (IPR000719; PF00069), 85 a protein tyrosine kinase domain (IPR001245; PF07714), and 64 a casein kinase-like domain (PTHR11909). Other assigned domains included the protein–protein interaction domains SH2 (IPR000980; SSF55550, *n* = 21) and SH3 (IPR001452; SSF50044; *n* = 10), as well as “fibronectin type III” (IPR003961; SSF49265; *n* = 18), “armadillo-type fold” (IPR016024; *n* = 17), and “immunoglobulin-like” domains (IPR007110; IPR013098; PF07679; SSF48726; *n* = 14), all of which are accessory domains frequently found in protein kinase sequences (Manning *et al.* 2002b).

**Figure 2:**
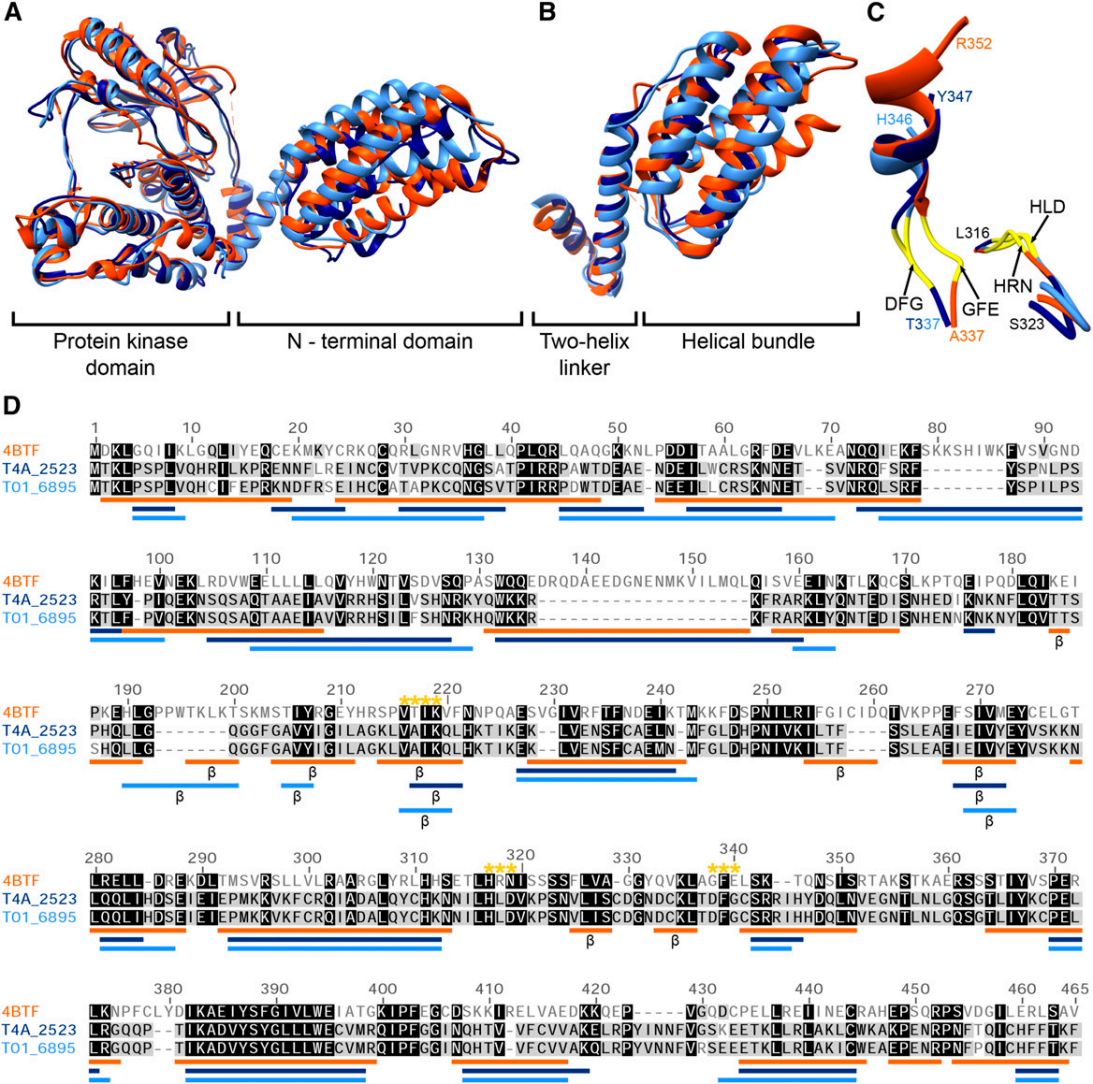
A novel, MOS-like protein kinase of *Trichinella spiralis* and *T. pseudospiralis*, which shares structural homology with the N-terminus of the murine mixed lineage kinase domain-like protein (MLKL). (A) Three-dimensional models of *T. spiralis* (T01_6895; light blue) and *T. pseudospiralis* (T4A_2523; dark blue) kinases superimposed on to the PDB (Protein Data Bank) structure of MLKL (4BTF; orange). (B) N-terminal domain comprised of a two-helix linker and a helical bundle. (C) Superimposed catalytic cleft of 4BTF, T01_6895, and T4A_2523. Motif regions are annotated and shown in yellow. Positions and amino acids of the last residues shown are indicated in the respective color for each model (black if the residues are the same in all three models). Numbering of residues is based on the multiple sequence alignment. (D) Multiple sequence alignment showing levels of similarity among residues for all three sequences (white to black; not conserved to conserved). Secondary structures based on three-dimensional modeling using the program I-TASSER for *Trichinella* models and based on the 4BTF crystal structure are depicted below the alignment; α helices are represented by lines in the same color as the respective model and beta sheets are also marked with a “β”. Motifs essential for kinase activity are marked with yellow stars.

In addition to these domains, we identified a pair of kinase sequences (T01_6895 and T4A_2523) in the “Other” group that could not be classified and/or annotated further, but had been proposed previously (Desjardins *et al.* 2013) to represent a novel kinase in *T. spiralis* (Tsp_04914). Their amino acid sequences did not match any domains other than the kinase catalytic domain (PF00069), which shared some sequence similarity with that of MOS kinases in a range of eukaryotes (90–100% with other *Trichinella* species, 55–56% with *Trichuris* species, and 34–43% with various other metazoans). This is the first report of MOS-like kinase catalytic domains for any nematode. However, the N-terminal regions of these kinases were substantially longer than those of all known MOS kinases in KinBase and NCBI-nr (with the exception of one sequence from the sea urchin *Strongylocentrotus purpuratus*; XP_003729407) and did not match sequences of any taxa other than those of the class Enoplea (*i.e.*, *Trichinella* and *Trichuris* spp.; NCBI-nr BLASTp, E-value < 2.5 and NCBI-nr BLASTn, E-value < 10^−5^). This finding stimulated us to investigate these unclassified kinase sequences further by subjecting their inferred sequences to structural modeling. The predicted protein structures shared highest structural similarity with that of a mixed lineage kinase domain-like protein of mice (MLKL; Protein Data Bank identifier: 4BTF), with root-mean-square deviation values and TM-scores of 6.6 and 0.73, and 6.4 and 0.74, for *T. spiralis* and *T. pseudospiralis*, respectively (Figure 2A). In these two models, the N-terminal region, despite sharing low amino acid sequence identity (8.5–16.7%) and similarity (16.7–29.8%) to the MLKL N-terminus, assumed the same fold as this region of MLKL, forming a helical bundle and a two-helix linker (Figure 2B; *cf*. Murphy *et al.* 2013). To explore this aspect further and to exclude the possibility that any protein kinase with a similarly-sized (≥ 150 aa) N-terminal region would model to the MLKL structure, we applied I-TASSER to a MOS kinase from the sea urchin, *S. purpuratus* (XP_003729407), which has a 150 amino acid-long N-terminus. However, we were not able to model a three-dimensional structure for this N-terminal region (not shown). When we modeled the mouse MOS kinase sequence (P00536.2), it had most structural similarity to the human feline sarcoma viral oncogene homolog (v-FES; 3BKB). Taken together, these results suggest that the two novel, MOS-like kinases, T01_6895 and T4A_2523, are structurally distinct from known MOS kinases.

A detailed appraisal of both *Trichinella* kinase sequences revealed three conserved motifs, VAIK, HLD, and DFG, suggesting that they are active kinases (Figure 2C), consistent with the finding that their catalytic domains share most sequence homology with that of MOS kinases. In contrast, the motifs HLD and DFG are not conserved in the pseudokinase MLKL (Murphy *et al.* 2013; Figure 2, C and D). An amino acid sequence alignment of the complete sequences revealed 87 additional motifs or residues that were conserved among mouse, *T. spiralis*, and *T. pseudospiralis* (Figure 2D); also the secondary structures predicted from these sequences were similar among all three species (Figure 2D).

## Discussion

The reversible phosphorylation of proteins by protein kinases is an essential biochemical process, and is relatively conserved in eukaryotes. However, some kinase groups have evolved to assume new functions in some invertebrate groups (Manning *et al.* 2002a; Manning 2005). While several major kinase expansions have been reported for the free-living nematode *C. elegans* (see Manning *et al.* 2002a; Manning 2005), there is no information for the vast majority of other members of the phylum Nematoda. As some nematode groups vary considerably in their biology and life cycles, this variability might be reflected in protein kinase complements. Here, we curated and explored in detail the kinomes of representatives of the *Trichinella* complex, which assume a unique taxonomic position (class Enoplea) within the Nematoda compared with other parasitic and free-living nematodes (class Chromadorea; Blaxter *et al.* 1998; Roberts and Janovy 2009; Blaxter and Koutsovoulos 2015; Korhonen *et al.* 2016). In this context, we defined, curated, and compared the kinomes of two of the most-studied species within this complex, *T. spiralis* and *T. pseudospiralis* (see Pozio and Darwin Murrell 2006; Pozio and Zarlenga 2013), to provide a basis for quantitative and qualitative comparisons of their kinomes with other nematode species and also for exploring cellular signaling mechanisms in these parasites in the future.

The application of our bioinformatic pipeline enabled us to curate and/or improve gene predictions for 32 of 226 (*T. spiralis*; 14%) and 60 of 232 (*T. pseudospiralis*; 26%) protein kinase genes, respectively. We reveal that *T. spiralis* and *T. pseudospiralis* have remarkably “compact” kinomes, the smallest curated metazoan kinomes to date. The latter statement is supported by comparisons with draft kinomes (suggested to contain 234–364 kinases) of six other nematodes (*Ascaris suum*, *Brugia malayi*, *Loa loa*, *Meloidogyne hapla*, *Pristionchus pacificus*, and *Wuchereria bancrofti*; *cf*. Desjardins *et al.* 2013) and curated kinomes of other metazoans (vinegar fly, mouse, and sea urchin; *cf*. KinBase). Although there is a possibility that the kinase complements inferred for *Trichinella* might be slightly underestimated, because of a lack of transcriptomes for all developmental stages of these taxa and/or an inability to identify and classify orphan (unknown) kinases, we offer a number of explanations for these “reduced” kinomes. First, *Trichinella* could have developed an efficient way of controlling intra- and intercellular signaling that is distinct from other metazoans. Second, it is conceivable that *Trichinella* species rely heavily on host cell pathways for particular metabolic processes and have thus lost kinase genes and/or other genes involved in such processes. This proposal is supported by some evidence for other parasitic worms representing nematodes, trematodes, and cestodes for which data are available (Tsai *et al.* 2013; Tyagi *et al.* 2015), and by the finding that only eight kinases (representing the seven groups Atypical, CK1, CMGC, Other, RGC, STE, and TKL) are linked to metabolism (KEGG category) in *Trichinella*. In contrast, there was a considerably higher number of kinases (RGC group) in this KEGG category in both the extracellular, parasitic nematode *H. contortus* (*n* = 13; *cf*. Stroehlein *et al.* 2015) and the free-living nematode *C. elegans* (*n* = 23). Whether the lack of kinases, such as RAD53, NEK, and TTK, which are principally associated with nonmetabolic pathways, can also be attributed to gene loss or simply to an efficient use of alternative or novel pathways, is presently unclear. However, the fact that kinases of the RAD53, NEK, and TTK subfamilies play roles in the control of the cell cycle, including arrest upon DNA damage (Pellicioli and Foiani 2005; Fry *et al.* 2012; Liu and Winey 2012), is an example that supports the latter hypothesis, and suggests that, for *Trichinella*, these mechanisms are regulated by efficient signaling cascades involving small numbers of kinases. Other kinases not encoded in *Trichinella*, such as the growth factor receptors VEGFR and PDGFR, are also absent from all other nematodes and flatworms studied to date. Given that VEGFR-encoding genes are present in the kinomes of human, mouse, sea urchin, and vinegar fly (http://kinase.com/web/current/kinbase/genes/Family/VEGFR/),which represent both deuterostomes and protostomes, it is plausible that these kinases have been lost from both the phyla Nematoda and Platyhelminthes during evolution.

The reduced kinomes of *T. spiralis* and *T. pseudospiralis* might also be partially explained by the unique biology of these species with respect to other nematodes. For instance, the small number of RGCs (*n* = 3) in *Trichinella* compares with an expansion of these cyclases in nematodes, such as *C. elegans* (*n* = 27) and *H. contortus* (*n* = 28), with a free-living phase in their life cycle, where these kinases play important roles in environmental sensing (Ortiz *et al.* 2009; Adachi *et al.* 2010). This finding supports the proposal that the small RGC group in *Trichinella* can be attributed to the unique biology of this species. Nevertheless, as the L1 stage of *Trichinella* actively interacts with its intracellular environment (Despommier 1998; Smith *et al.* 2000), it is possible that some of the 66 protein kinases inferred to be associated with the processing of environmental cues (*cf*. Table S1 and Table S2) are involved in sensing. Although it is not yet clear which of these kinases are involved in pathways linked to sensing, they are distinct from RGCs used by other nematodes and might be those within groups AGC, TK, STE, and CAMK.

The small number of kinases in most families and subfamilies (the majority being represented by a single kinase), and the finding that > 90% of all identified kinases were present as single-copy orthologs, further support the notion of a functionally efficient kinome. One notable exception is the TTBK family, with 19 and 23 members in *T. spiralis* and *T. pseudospiralis*, respectively. Although a TTBK-like family of 31 and 16 kinases has been defined for *C. elegans* and *H. contortus*, respectively (Manning 2005; Stroehlein *et al.* 2015), these are distinct from the recognized TTBK family for *Trichinella*. This information suggests a distinctiveness of function for TTBK kinases in *Trichinella* compared with other nematodes.

A comparison of the kinomes of the encapsulated (*T. spiralis*) and nonencapsulated (*T. pseudospiralis*) taxa indicates that the biological distinctiveness of these species cannot be attributed to kinase signaling alone, given that we did not detect any major differences in the kinase complement between the two species. However, we did detect three differences in the numbers of encoded kinases (Figure 1, Table S1, and Table S2), suggesting that *T. pseudospiralis* has multiple copies of kinase-encoding genes in the groups CK1 and TK (FER family). Although the present data sets did not allow us to unambiguously verify these apparent gene duplication events in *T. pseudospiralis*, long-read sequencing technologies (Roberts *et al.* 2013) should enable genomes of chromosome-level contiguity to be assembled for *T. spiralis*, *T. pseudospiralis*, and all other *Trichinella* taxa in the future. This effort should enable intertaxon comparisons of these genes and assist in establishing whether these differences are linked to the unique biology of nonencapsulated *vs.* encapsulated taxa.

Despite the high, overall similarity of the kinomes of the two *Trichinella* species, their kinase complements were considerably different from those of other parasitic and free-living nematodes. Although current evidence suggests that a small complement of kinases (∼235–250) is typical of enopleans (*cf*. Schiffer *et al.* 2013; Jex *et al.* 2014), further work is required to confirm this proposal. Here, we also provided support that most of the nematode-specific “Worm” families are indeed *Caenorhabditis*-specific (Figure S2, Table S3, Table S4, Table S5, Table S6, Table S7, Table S8, Table S9, and Table S10), with the exception of the WORM6 family, which is also found in *Trichinella* and other nematodes (Desjardins *et al.* 2013; Stroehlein *et al.* 2015). In contrast, the small size of the FER family seems to be restricted to *Trichinella* and also to *M. hapla* (see Desjardins *et al.* 2013), with a larger FER family encoded in some filarial and rhabditid nematodes (Desjardins *et al.* 2013).

The classification of protein kinases based on primary sequence similarity in the catalytic domain and/or accessory domains enabled us to discern differences in the numbers of representatives within families/subfamilies. However, in some instances, functional annotation was compromised because of very limited amino acid sequence similarity (particularly external to the catalytic domain) to *C. elegans* and *H. sapiens* homologs for which experimental and functional data are available (*cf*. Figure S2, Table S3, Table S4, Table S5, Table S6, Table S7, Table S8, Table S9, and Table S10). Therefore, we used a three-dimensional modeling approach (employing the program I-TASSER) to infer structural homology of one such amino acid sequence, allowing statistically-supported functional annotation (Zhang and Skolnick 2004).

The presence of an N-terminal helical bundle and linker tethered to the kinase domain of a previously unclassified kinase (*i.e.*, without family or subfamily classification within the “Other” group) in *T. spiralis* and *T. pseudospiralis* suggests that this kinase assumes a pore-forming and membrane-permeabilizing function, as has been reported for murine and human mixed-lineage kinase domain-like proteins (MLKLs) that also have this N-terminal domain (Murphy *et al.* 2013; Hildebrand *et al.* 2014; Su *et al.* 2014; Wang *et al.* 2014). Whether the N-terminus of MOS-like kinases in *Trichinella* plays a functional role in tumor necrosis factor-induced cell death (necroptosis), as MLKLs do in chordates (Newton and Manning 2016), is not yet known. Homologs of the upstream kinases, RIP1 and RIP3, which form a complex called “necrosome” together with MLKL (Murphy *et al.* 2013), are absent from both *T. spiralis* and *T. pseudospiralis*. In mice, phosphorylation by RIP3 is required for the activation of MLKL and for membrane localization (Sun *et al.* 2012; Murphy *et al.* 2013). However, it is conceivable that, instead of RIP3, one or more serine-threonine protein kinases encoded in the *Trichinella* genomes might phosphorylate and activate these two novel kinases.

Sequence conservation in all motifs linked to kinase activity, as well as sequence similarity to the catalytic domain of MOS kinases, raises the question as to whether the MOS-like kinases of *Trichinella* are capable of (auto-)phosphorylation. Although structural similarity in their N-terminus suggests MLKL kinase-like function (Newton and Manning 2016), they might also act like MOS kinases (*i.e.*, in oocyte maturation; Gebauer and Richter 1997; Sagata 1997; Dupré *et al.* 2011). However, it is also possible that they assume a role that is distinctive from those of both MOS and MLKL proteins. The very low transcription levels of MOS-like kinase genes in the L1 stages of *T. spiralis* and *T. pseudospiralis* (see Korhonen *et al.* 2016) indicate that they are unlikely to play a major role in host-parasite interactions while inside the host cell. A preliminary study of *Trichuris suis*, a parasitic nematode in the same class (Enoplea) as members of the *Trichinella* complex, has shown that a MOS-like kinase gene is almost exclusively transcribed in the posterior part of the adult female worm (Jex *et al.* 2014), which hints to a possible role in reproductive processes. While in mice MLKL does not appear to play a major role in reproduction, with MLKL-deficient mice being viable and without an overt phenotype (Murphy *et al.* 2013; Wu *et al.* 2013), another study (Blohberger *et al.* 2015) showed that the necroptosis pathway is involved in the regulation of cell death in human ovarian cells. Additionally, the catalytic domains of *Trichinella* MOS-like kinases share more sequence similarity with those of MOS kinases than those of MLKL kinases; given that MOS plays a key role in the meiotic maturation of oocytes (Gebauer and Richter 1997; Sagata 1997; Dupré *et al.* 2011), these kinases might be involved in reproductive or developmental processes in *Trichinella*. Clearly, future investigations should explore the functional roles of these interesting and apparently enoplean-specific kinases.

In conclusion, the present study provides first detailed information on the kinomes of *T. spiralis* and *T. pseudospiralis*, as well as a useful, new resource for comparative studies of nematode kinomes. We also report an example of the utility of three-dimensional modeling for the functional annotation of kinases previously unannotatable using conventional bioinformatic techniques. Taken together, the present results provide a foundation for comparative studies of nematode kinomes and their evolution, and might also enable the identification of enoplean-specific intervention and diagnostic targets. Significantly, the *in silico*-modeling approach evaluated here might pave the way to being able to identify and classify presently unknown (orphan) kinases in parasitic worms for subsequent structural and functional investigations.

## Supplementary Material

Supplemental Material
