# Supplementary material for: Analyses of Compact Trichinella Kinomes Reveal a MOS-Like Protein Kinase with a Unique N-Terminal Domain
Source: G3 (Bethesda). 2016 Jul 13;6(9):2847–56. doi: 10.1534/g3.116.032961 (PMC5015942; doi:10.1534/g3.116.032961)
Supplement: Supplemental Material [file supp_6_9_2847__index.html]

Analyses of Compact Trichinella Kinomes Reveal a MOS-Like Protein Kinase with a Unique N-Terminal Domain — Supplemental Material 

# Analyses of Compact *Trichinella* Kinomes Reveal a MOS-Like Protein Kinase with a Unique N-Terminal Domain

## Supplemental Material for Stroehlein, *et al*, 2016

**Files in this Data Supplement:**

- Figure S1 - Trees representing the phylogenetic relationship of eukaryotic protein kinase (ePK) sequences between *Trichinella spiralis* (T1) and *T. pseudospiralis* (T4A). Each ePK group is represented by an individual tree (A-I). Nodal support values (Bayesian inference) and sequence identifiers are given at the nodes and tips, respectively. (.pdf, 207 KB)
- Figure S2 - Clusters of orthologs among *Trichinella spiralis* (T1), *T. pseudospiralis* (T4.1), *Caenorhabditis elegans* (CEL) and *Homo sapiens* (HSA) based on orthoMCL clustering (E-value ≤ 1e-5; similarity ≥ 0.8). Individual sequence identifiers are given in Tables S3-S10. (.pdf, 61 KB)
- Table S1 - The *Trichinella spiralis* (T1) kinome, orthologs in *T. pseudospiralis* (T4.1), amino acid sequence identities and similarities, excretory/secretory prediction, functional annotations, and amino acid sequences. (.xlsx, 185 KB)
- Table S2 - The *Trichinella pseudospiralis* (T4.1) kinome, orthologs in *T. spiralis* (T1), amino acid sequence identities and similarities, excretory/secretory prediction, functional annotations, and amino acid sequences. (.xlsx, 216 KB)
- Table S3 - Clusters of orthologs among *Trichinella spiralis* (T1), *T. pseudospiralis* (T4.1), *Caenorhabditis elegans* (CEL) and *Homo sapiens* (HSA). (.xlsx, 13 KB)
- Table S4 - Clusters of orthologs among *Trichinella spiralis* (T1), *T. pseudospiralis* (T4.1) and *Caenorhabditis elegans* (CEL). (.xlsx, 36 KB)
- Table S5 - Clusters of orthologs among *Trichinella spiralis* (T1), *T. pseudospiralis* (T4.1) and *Homo sapiens* (HSA). (.xlsx, 44 KB)
- Table S6 - Clusters of orthologs between *Trichinella spiralis* (T1) and *T. pseudospiralis* (T4.1). (.xlsx, 22 KB)
- Table S7 - *Trichinella pseudospiralis* (T4.1) sequences without orthologs in other species. (.xlsx, 9 KB)
- Table S8 - Clusters of orthologs between *Caenorhabditis elegans* (CEL) and *Homo sapiens* (HSA). (.xlsx, 26 KB)
- Table S9 - *Caenorhabditis elegans* (CEL) sequences without orthologs in other species. (.xlsx, 125 KB)
- Table S10 - *Homo sapiens* (HSA) sequences without orthologs in other species. (.xlsx, 125 KB)
