# Supplementary material for: Analyses of Compact Trichinella Kinomes Reveal a MOS-Like Protein Kinase with a Unique N-Terminal Domain
Source: G3 (Bethesda). 2016 Jul 13;6(9):2847–56. doi: 10.1534/g3.116.032961 (PMC5015942; doi:10.1534/g3.116.032961)
Supplement: Supplemental Material [file supp_g3.116.032961_FigureS1.pdf]

Supplementary File 1

Analyses of Compact *Trichinella* Kinomes Reveal a MOS-like Protein Kinase with a Unique N-terminal Domain

Andreas J. Stroehlein*, Neil D. Young*, Pasi K. Korhonen*, Bill C.H. Chang*[,†], Paul W. Sternberg[‡], Giuseppe La Rosa[§], Edoardo Pozio[§] and Robin B. Gasser*[,1]

*Faculty of Veterinary and Agricultural Sciences, The University of Melbourne, Parkville, Victoria 3010, Australia

[†]Yourgene Bioscience, Shu-Lin District, New Taipei City 23863, Taiwan

[‡]Division of Biology, HHMI, California Institute of Technology, Pasadena, California 91125, USA

[§]Istituto Superiore di Sanità, 00161 Rome, Italy

Figure S1 Trees representing the phylogenetic relationship of eukaryotic protein kinase (ePK) sequences between *Trichinella spiralis* (T1) and *T. pseudospiralis* (T4A). Each ePK group is represented by an individual tree (**A-I**). Nodal support values (Bayesian inference) and sequence identifiers are given at the nodes and tips, respectively

S1 A Protein kinases A, G and C, and other nucleoside-regulated kinases (AGC group)

S1 B Ca^{2+}/calmodulin-dependent kinases (CAMK group)

S1 C Casein kinase 1 (CK1 group)

S1 D Cyclin-dependent kinases (CDKs), mitogen-activated protein kinases (MAPKs), glycogen synthase kinases (GSKs) and CDK-like kinases (CMGC group)

S1 E “Other” protein kinases (Other group)

S1 F Receptor guanylate cyclases (RGC group)

S1 G MAPK cascade kinases (STE group)

S1 H Tyrosine kinases (TK group)

S1 I Tyrosine kinase-like kinases (TKL group)

Figure S1 A
AGC group

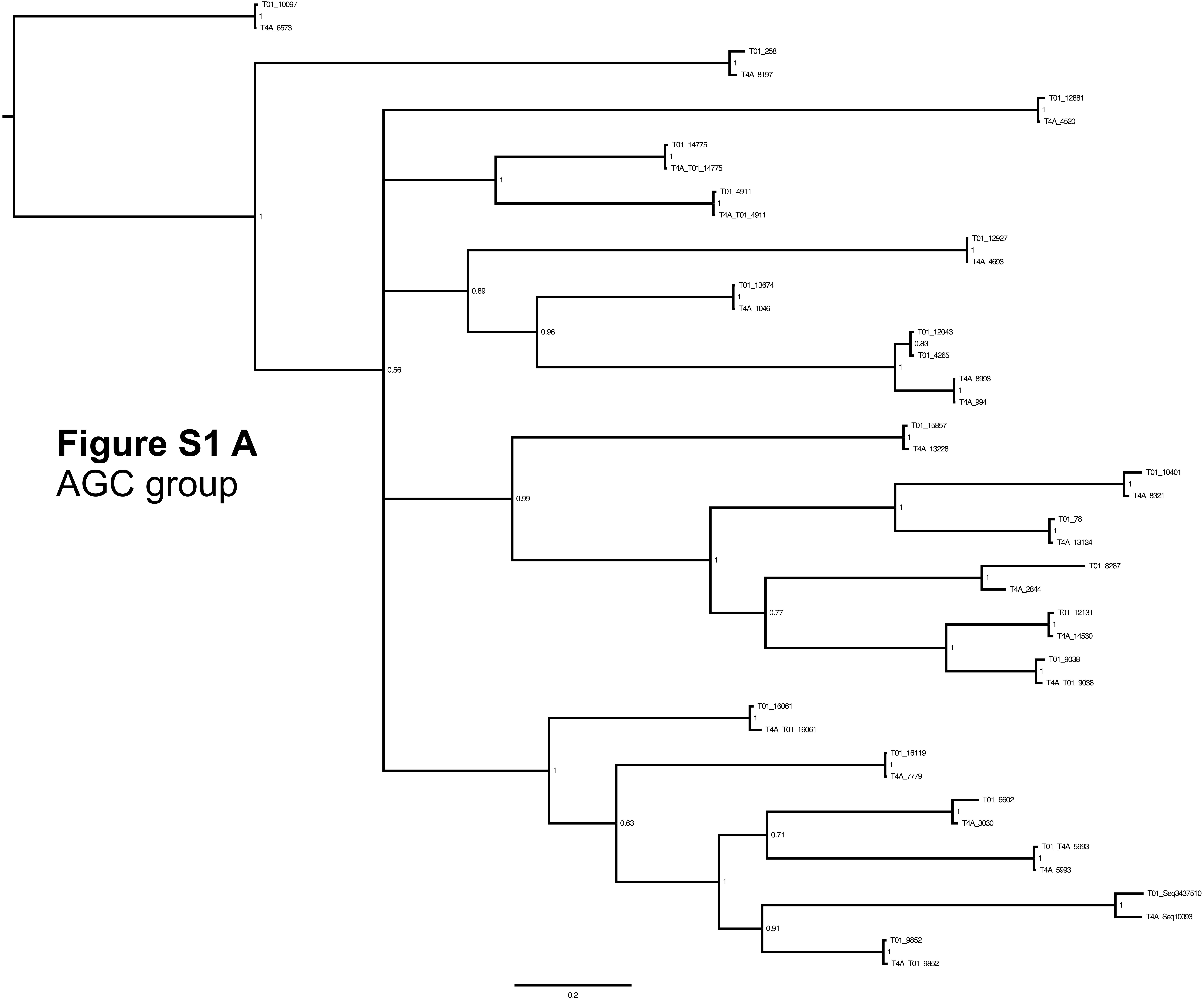

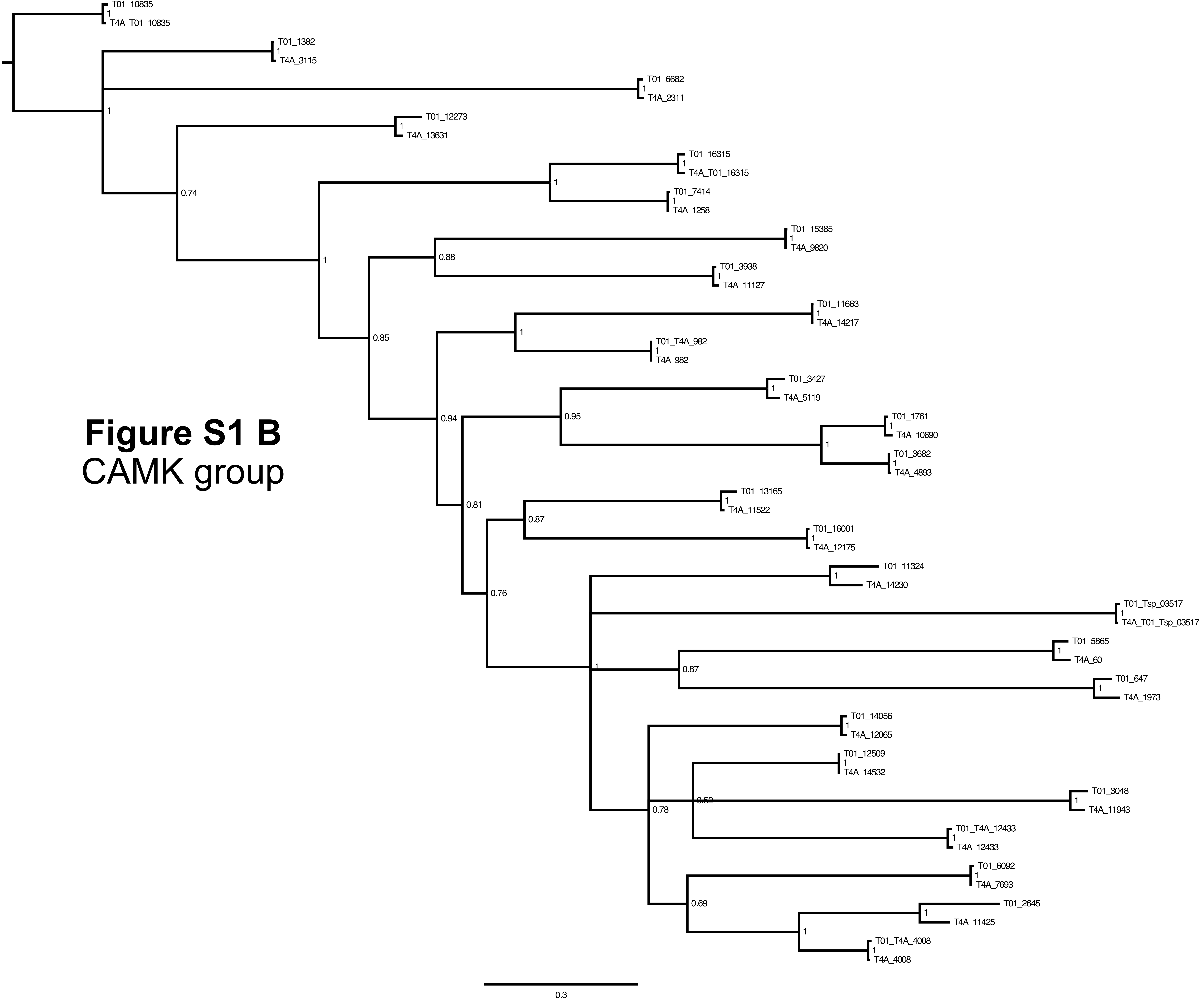

Figure S1 B
CAMK group

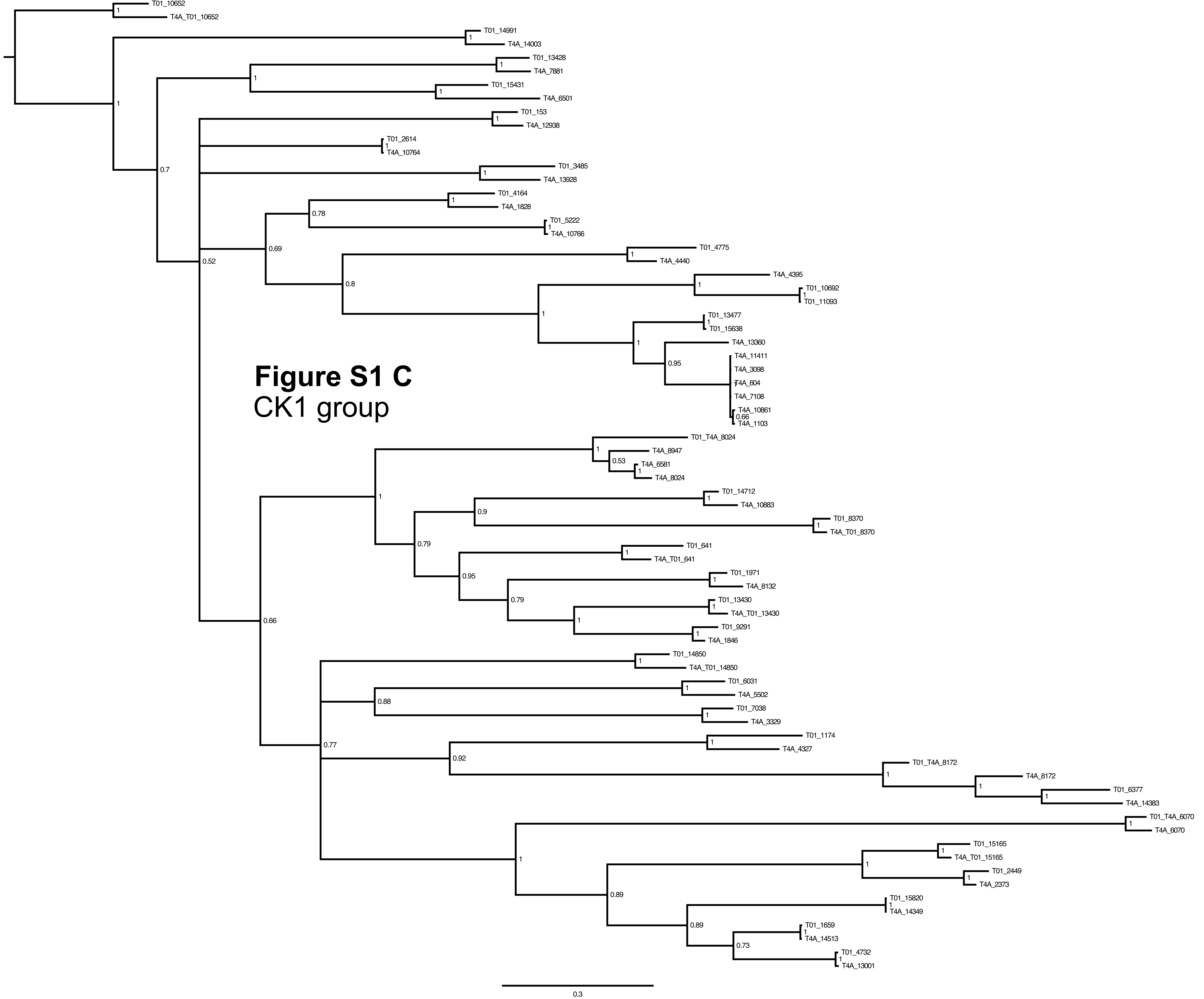

Figure S1 C
CK1 group

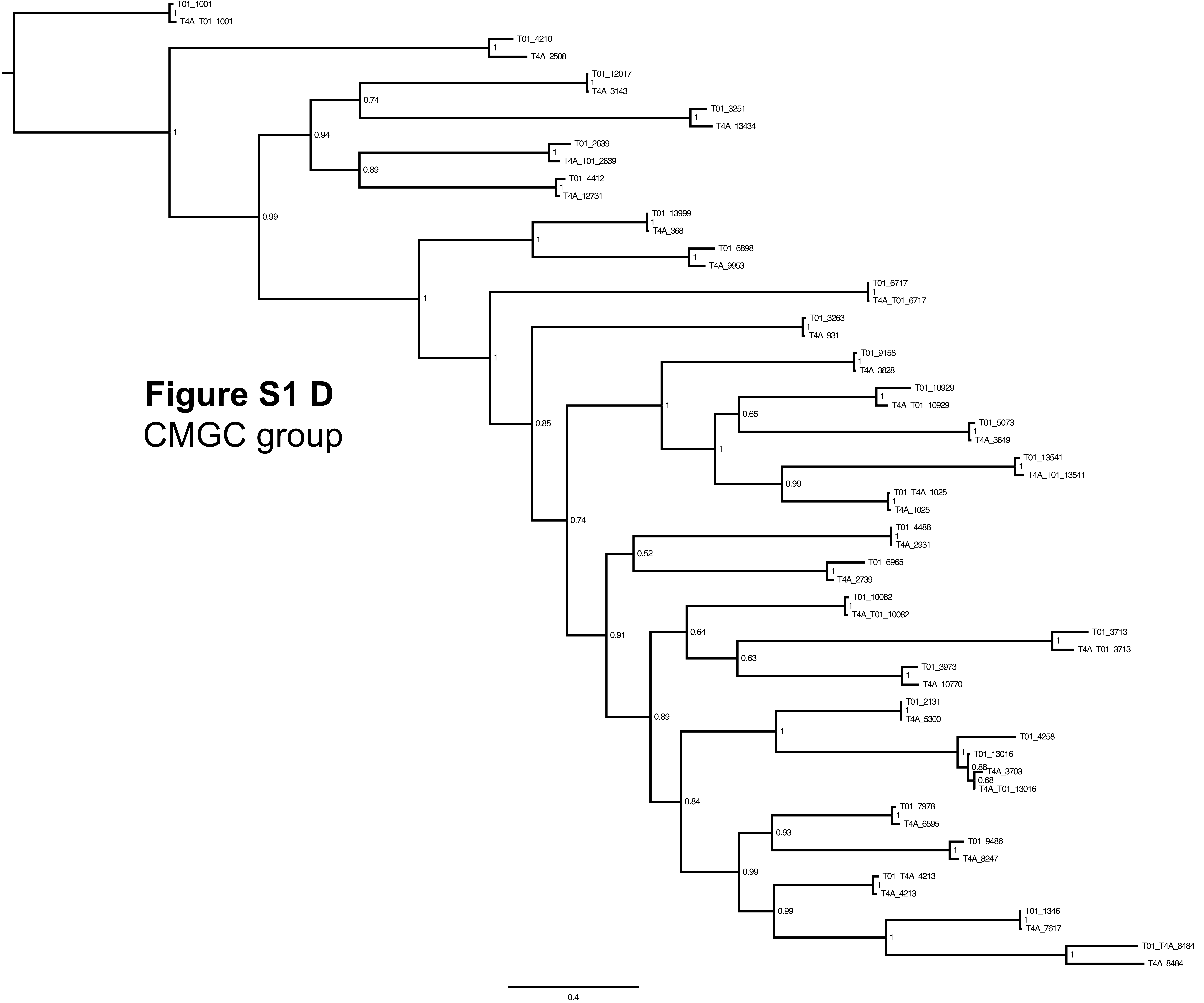

Figure S1 D
CMGC group

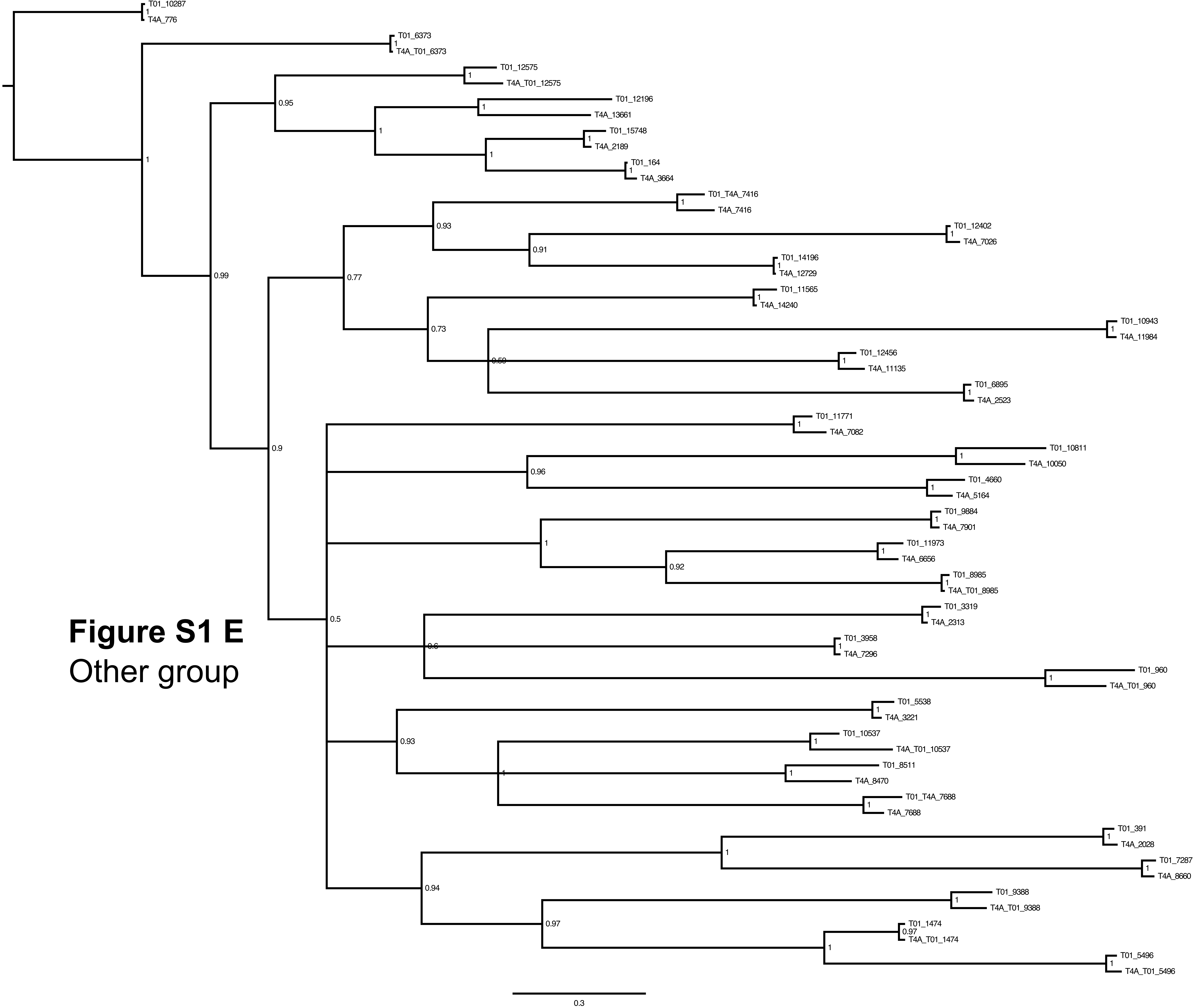

Figure S1 E
Other group

Figure S1 F

RGC group

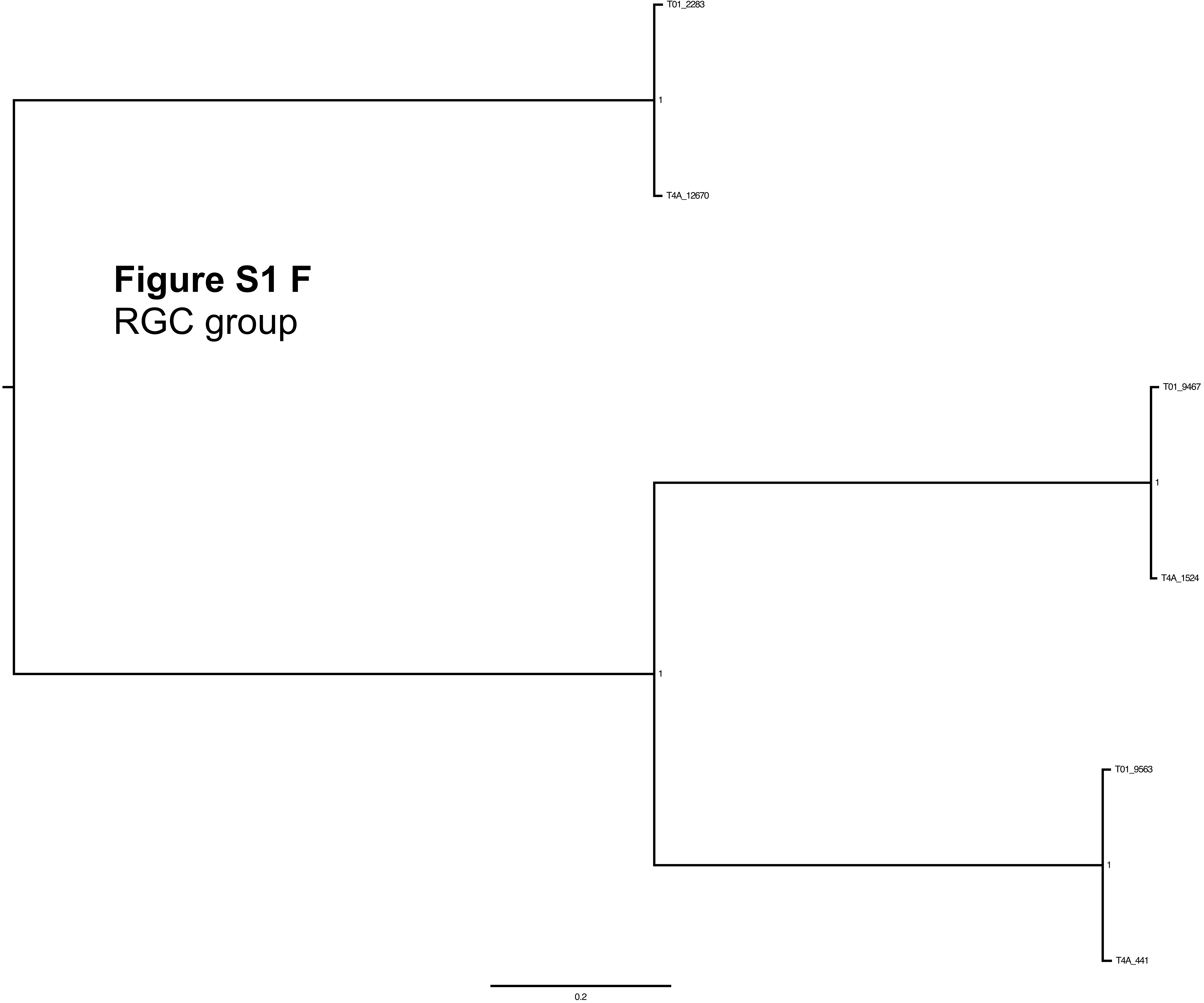

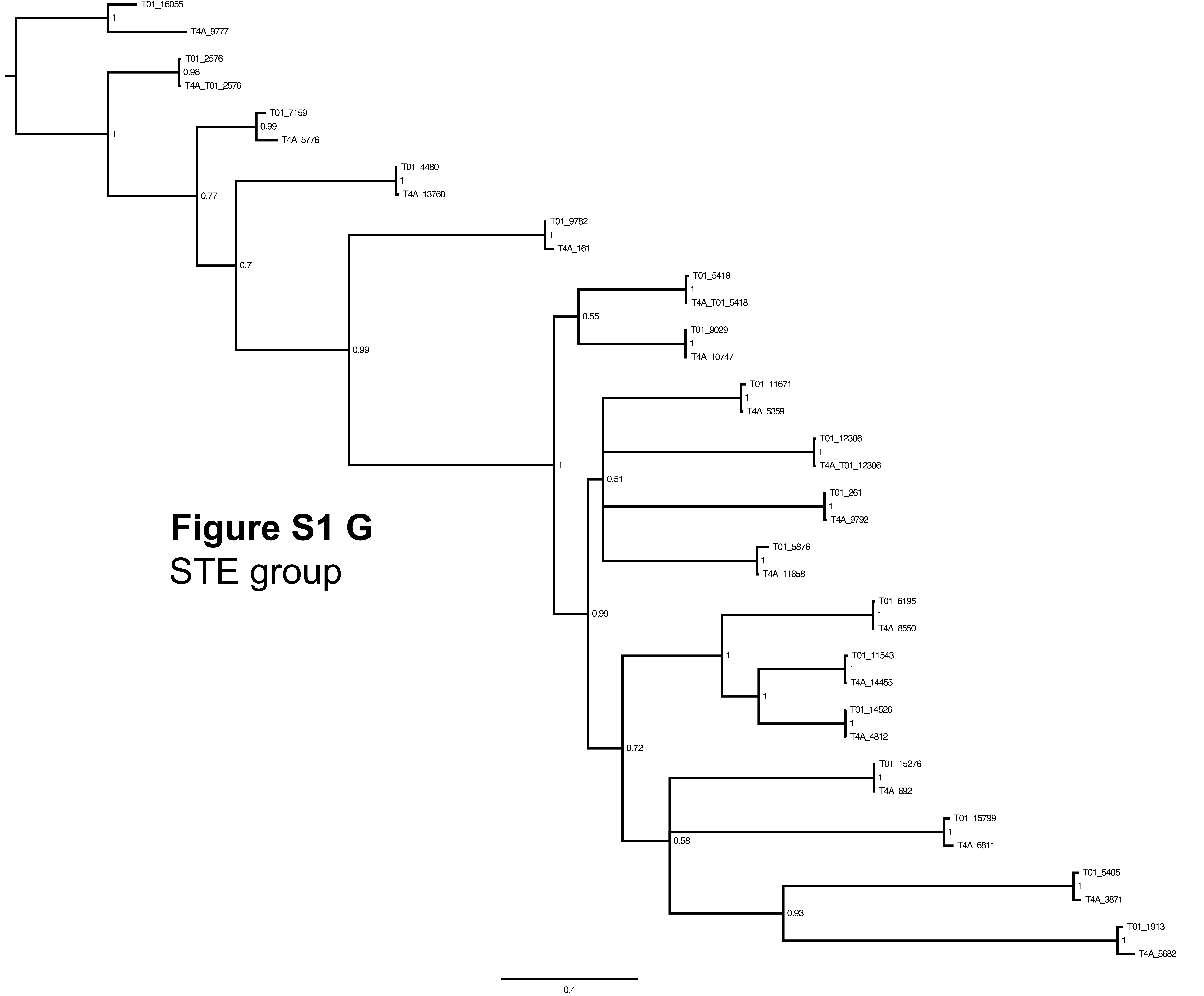

Figure S1 G
STE group

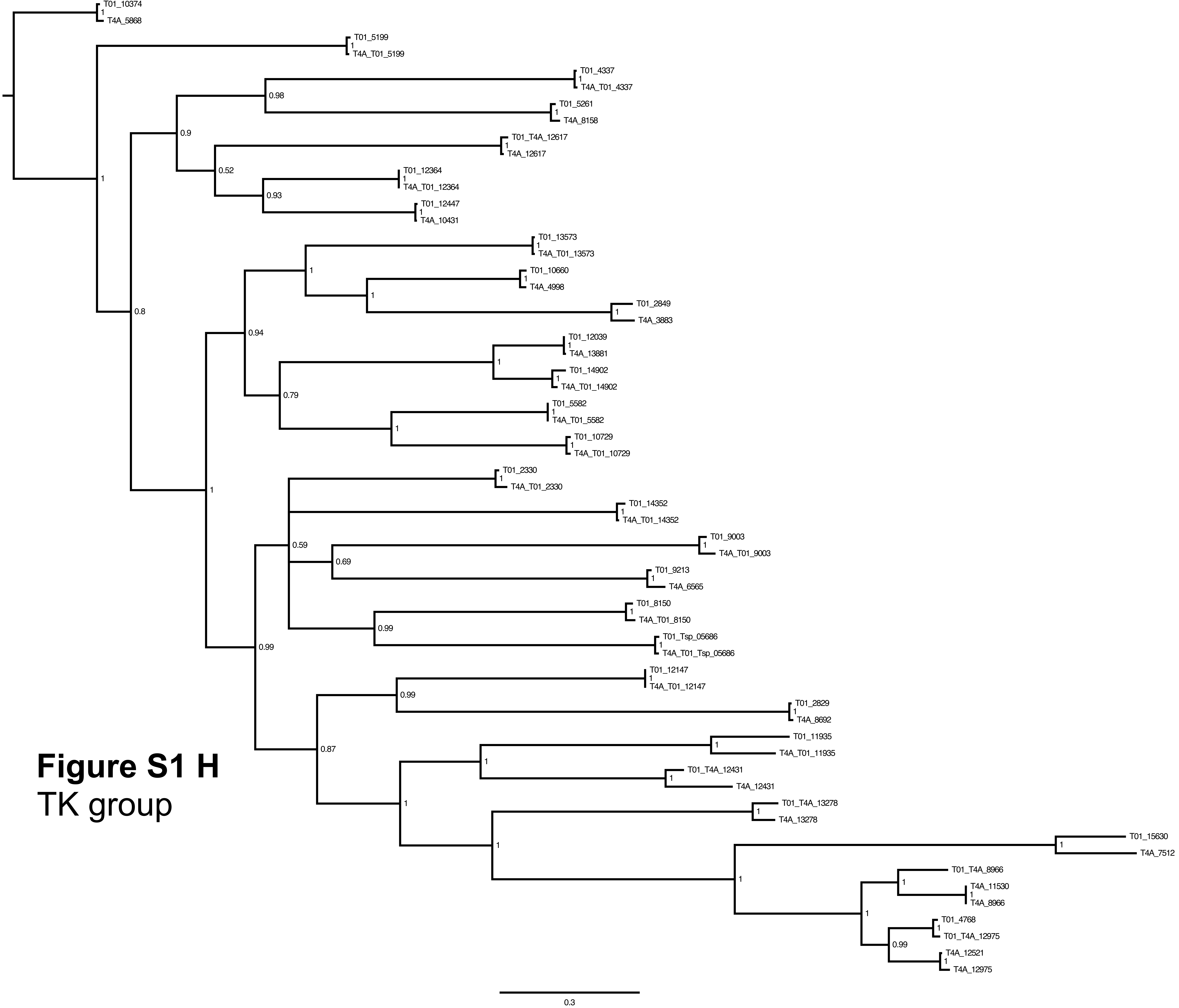

Figure S1 H
TK group

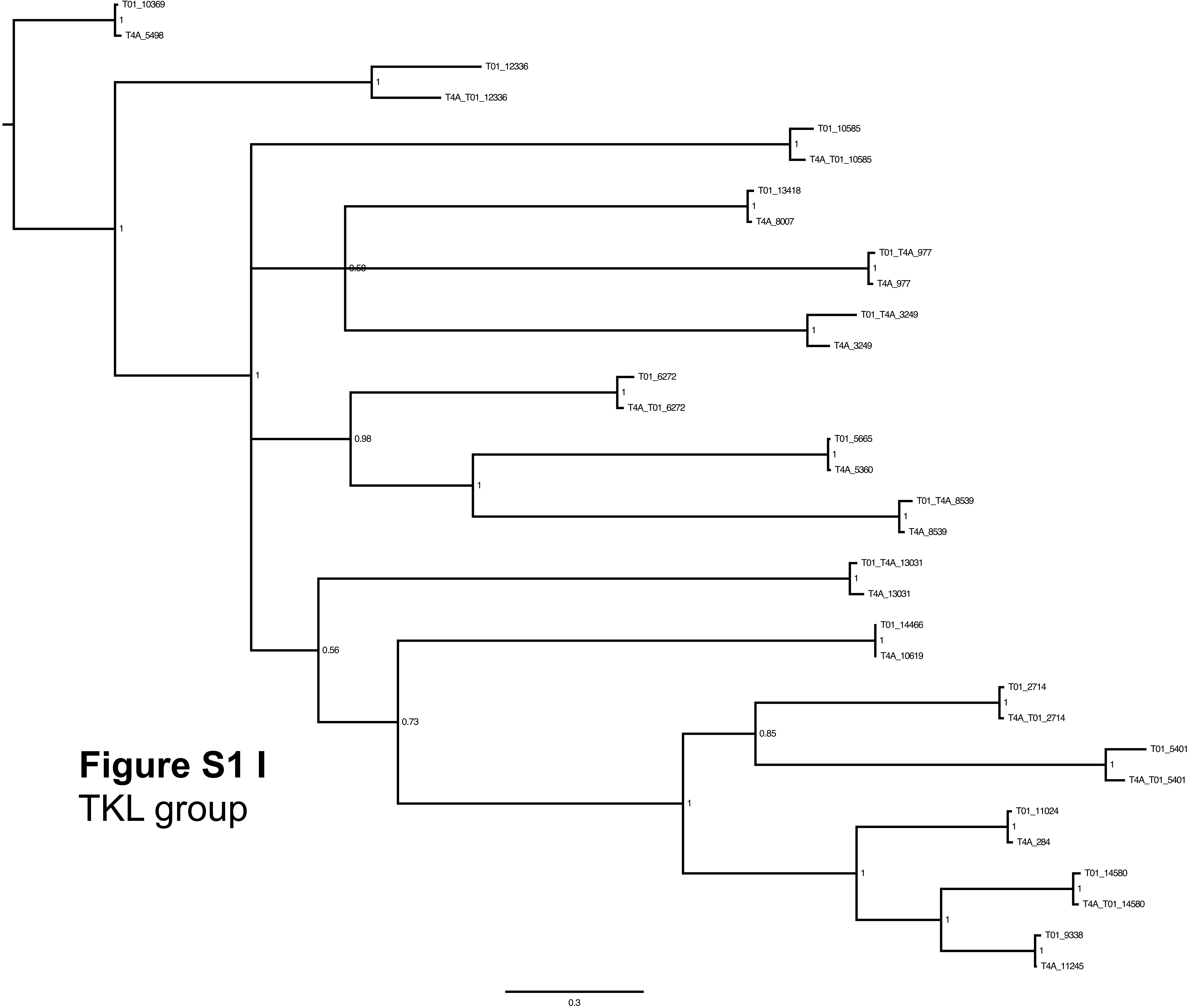

Figure S1 I
TKL group
