# Supplementary material for: Analyses of Compact Trichinella Kinomes Reveal a MOS-Like Protein Kinase with a Unique N-Terminal Domain
Source: G3 (Bethesda). 2016 Jul 13;6(9):2847–56. doi: 10.1534/g3.116.032961 (PMC5015942; doi:10.1534/g3.116.032961)
Supplement: Supplemental Material [file supp_g3.116.032961_FigureS2.pdf]

G3 Genes | Genomes | Genetics

Supplementary File 2

Analyses of Compact *Trichinella* Kinomes Reveal a MOS-like Protein Kinase with a Unique N-terminal Domain

Andreas J. Stroehlein*, Neil D. Young*, Pasi K. Korhonen*, Bill C.H. Chang*,†, Paul W. Sternberg‡, Giuseppe La Rosa§, Edoardo Pozio§ and Robin B. Gasser*,1

*Faculty of Veterinary and Agricultural Sciences, The University of Melbourne, Parkville, Victoria 3010, Australia

†Yourgene Bioscience, Shu-Lin District, New Taipei City 23863, Taiwan

‡Division of Biology, HHMI, California Institute of Technology, Pasadena, California 91125, USA

§Istituto Superiore di Sanità, 00161 Rome, Italy

Figure S2 Clusters of orthologs among *Trichinella spiralis* (T1), *T. pseudospiralis* (T4.1), *Caenorhabditis elegans* (CEL) and *Homo sapiens* (HSA) based on orthoMCL clustering (E-value ≤ $1e^{-5}$; similarity ≥ 0.8). Individual sequence identifiers are given in Tables S3-S10

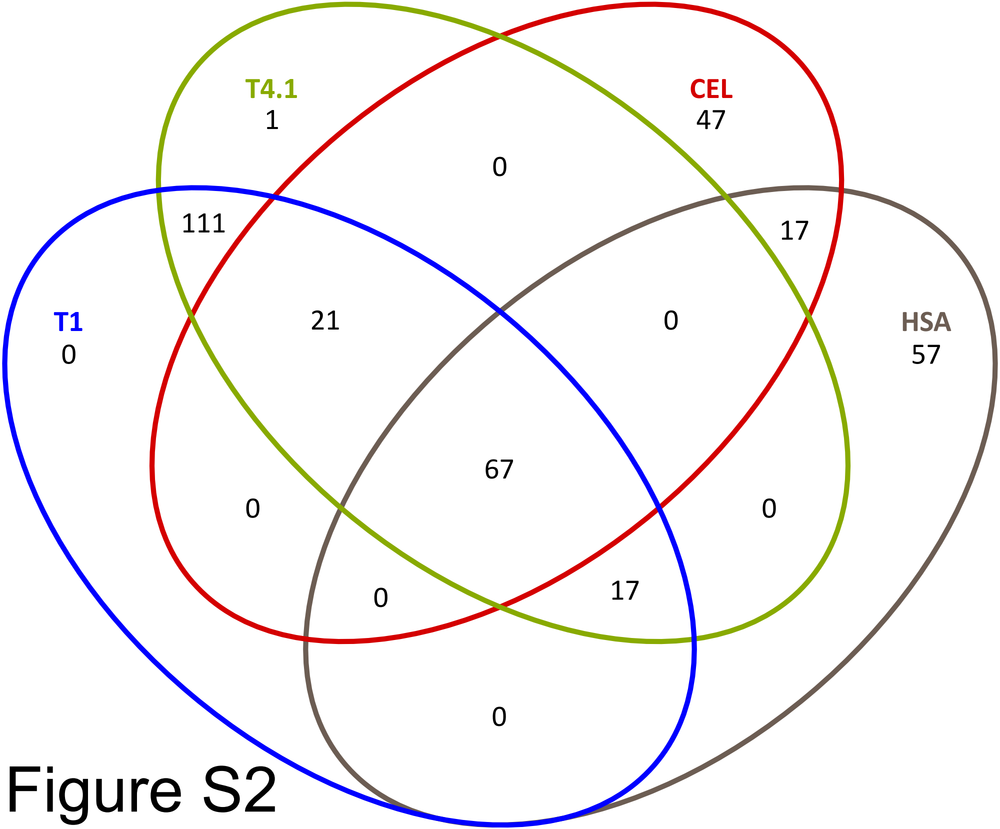

Figure S2
